# Adult-onset mitochondrial movement disorders: a national picture from the Italian Network

**DOI:** 10.1007/s00415-021-10697-1

**Published:** 2021-07-14

**Authors:** V. Montano, D. Orsucci, V. Carelli, C. La Morgia, M. L. Valentino, C. Lamperti, S. Marchet, O. Musumeci, A. Toscano, G. Primiano, F. M. Santorelli, C. Ticci, M. Filosto, A. Rubegni, T. Mongini, P. Tonin, S. Servidei, R. Ceravolo, G. Siciliano, Michelangelo Mancuso

**Affiliations:** 1 Department of Clinical and Experimental Medicine, Neurological Clinic, University of Pisa, Pisa, Italy; 2 Unit of Neurology, San Luca Hospital, Lucca, Italy; 3 IRCCS Istituto delle Scienze Neurologiche di Bologna, Programma di Neurogenetica, Bologna, Italy; 4 IRCCS Istituto delle Scienze Neurologiche di Bologna, UOC Clinica Neurologica, Bologna, Italy; 5 Dipartimento di Scienze Biomediche e Neuromotorie, Università di Bologna, Bologna, Italy; 6 UO Medical Genetics and Neurogenetics, Fondazione IRCCS Istituto Neurologico “Carlo Besta”, Milan, Italy; 7 Unit of Neurology and Neuromuscular Disorders, Department of Clinical and Experimental Medicine, University of Messina, Messina, Italy; 8 Fondazione Policlinico Universitario A. Gemelli, IRCCS, Rome, Italy; 9 Dipartimento di Neuroscienze, Università Cattolica del Sacro Cuore, Rome, Italy; 10 Molecular Medicine for Neurodegenerative and Neuromuscular Diseases Unit, IRCCS Stella Maris Foundation, Pisa, Italy; 11 Department of Clinical and Experimental Sciences, ASST Spedali Civili Brescia and NeMO-Brescia Clinical Center for Neuromuscular Diseases, University of Brescia, Brescia, Italy; 12 Department of Neurosciences, University of Torino, Turin, Italy; 13 Department of Neurosciences, Biomedicine and Movement Sciences, Section of Clinical Neurology, University of Verona, Verona, Italy

**Keywords:** Mitochondrial disorders, Ataxia, Parkinsonism, Movement disorders

## Abstract

**Introduction:**

Both prevalence and clinical features of the various movement disorders in adults with primary mitochondrial diseases are unknown.

**Methods:**

Based on the database of the “Nation-wide Italian Collaborative Network of Mitochondrial Diseases”, we reviewed the clinical, genetic, neuroimaging and neurophysiological data of adult patients with primary mitochondrial diseases (*n* = 764) where ataxia, myoclonus or other movement disorders were part of the clinical phenotype.

**Results:**

Ataxia, myoclonus and movement disorders were present in 105/764 adults (13.7%), with the onset coinciding or preceding the diagnosis of the mitochondrial disease in 49/105 (46.7%). Ataxia and parkinsonism were the most represented, with an overall prevalence at last follow-up of 59.1% and 30.5%, respectively. Hyperkinetic movement disorders were reported in 15.3% at last follow-up, being the less common reported movement disorders. The pathogenic m.8344A > G and *POLG* variants were always associated with a movement disorder, while LHON variants and mtDNA single deletions were more commonly found in the subjects who did not present a movement disorder. The most common neuroimaging features were cortical and/or cerebellar atrophy, white matter hyperintensities, basal ganglia abnormalities and nigro-striatal degeneration. Almost 70% of patients with parkinsonism responded to dopaminergic therapy, mainly levodopa, and 50% with myoclonus were successfully treated with levetiracetam.

**Conclusion:**

Movement disorders, mainly ataxia and parkinsonism, are important findings in adult primary mitochondrial diseases. This study underlies the importance of looking for a mitochondrial etiology in the diagnostic flowchart of a movement disorder and may help direct genetic screening in daily practice.

**Supplementary Information:**

The online version contains supplementary material available at 10.1007/s00415-021-10697-1.

## Introduction

Primary mitochondrial diseases (PMDs) are the most common group of metabolic inherited disease characterized by pathogenic variants in either the nuclear or mitochondrial genomes that directly or indirectly interfere with the respiratory chain function [1, 2]. PMDs are multisystemic disorders that mainly affect tissue requiring a high energy demand, including the central nervous system (CNS). Age of onset is variable and there are many clinical features of CNS involvement, among them ataxia, myoclonus and a broad spectrum of movement disorders [3]. In some of the mitochondrial phenotypes, ataxia or movement disorders are cardinal features (i.e., myoclonus or ataxia in MERRF, or ataxia in MIRAS), while in other PMDs, their prevalence is not well known, having been reported either anecdotally or in few cohorts [4, 5].

Moreover, little is known about genotype–phenotype correlation, clinical course, radiological features and response to therapy in mitochondrial movement disorders.

The aim of the present study was to characterize the cohort of Italian PMDs patient registered in the “Nationwide Italian Collaborative Network of Mitochondrial Diseases” affected by movement disorders.

**Table 1:** Phenotype

Phenotype	Predominant phenotype at baseline: number of patients (%)	Patients with this movement disorder as secondary features at onset	Patients developing this movement disorder later during the course of the disease	Cumulative prevalence at follow-up. Number of patients (%)
Ataxia	55 (53.9)	0	7	62 (59.1)
Hypokinetic	26 (24.8)	0	6	32 (30.5)
Myoclonus	13 (12.3)	6	3	22 (20.9)
Hyperkinetic	11 (10.5)	3	2	16 (15.3)

Prevalence of phenotype at baseline and last follow-up: percentages refers to proportions within the 105 patients who have a mitochondrial movement disorders

## Methods

We have selected from the Italian registry patients with the following movement disorders as a clinical manifestation of adult-onset (above age 16 years) PMD: ataxia/cerebellar signs, tremor, parkinsonism, dystonia, dyskinesias, and myoclonus. The Italian registry is the National platform where the different Italian centers collect detailed data of PMDs patients, in whom the diagnosis is confirmed either by the presence of a mtDNA or nuclear DNA pathogenetic variant or if the clinical picture, muscle biopsy and other tests strongly support PMD diagnosis.

The clinical section of the dataset includes dichotomous (“yes or no”) items that allowed us to subdivide the whole sample (as at the end of 2019) into two groups according to the presence or absence of movement disorders, and to analyze the main molecular features of each of these groups. Since the registry does not contain a specific dataset for a detailed characterization of the movement disorders features, an online 50-item Google Form^®^-based questionnaire was sent by email to all centers following adult PMDs (supplementary Table 1). The present study does not include patients with NARP, Leigh or other pediatric mitochondrial syndromes, that have movement disorders among the core clinical features [6], which are described separately [7].

The database establishment (and its use for scientific purposes) was approved by the local Ethical Committees of the single centers, which obtained written informed consent from all patients or their legal representatives and has been performed in accordance with the ethical standards laid down in the 1964 Declaration of Helsinki. This is a retrospective study; all the involved centers have specific expertise in PMDs.

We analyzed the main clinical, molecular, and radiological features from the 50-item Google Form^®^. Given that many patients presented a complex phenotype (i.e., a combination of two movement disorders at baseline, or an additional movement disorder during the course of the MD), for each patient the investigator defined at baseline the most relevant movement disorders (the predominant phenotype at baseline, Table 1) and the concomitant secondary features. Age at onset was referred to the onset of the predominant movement disorder. Disease onset was defined as the age of the clinical or molecular diagnosis of PMD. In addition to the predominant movement disorder, it was also assessed whether the patient developed other movement disorders at follow-up.

**Table 2:** Genotype with associated predominant phenotype at baseline

GENE	*N*	%	Predominant associated phenotype at baseline			
			Ataxia	Myoclonus	Hypokinetic	Hyperkinetic
*POLG1*	23 (12 AD, 11 AR)	22.0	16 (6 AD, 10 AR)	0	6 (5 AD, 1 AR)	1 (AD)
*MT-TK*	19	18.1	8	10	0	1
*Multiple mtDNA deletions*	10	9.5	1	0	6	3
*MT-TL1*	8	7.7	8	0	0	0
*Single mtDNA deletion*	9	8.6	3	0	3	3
*TWNK*	7	6.7	2	0	5	0
*OPA1*	4(*AD*)	3.8	2	0	2	0
*MT-TS1*	2	1.9	2	0	0	0
*AARS2*	1	0.9	1	0	0	0
*DARS2*	1	0.9	1	0	0	0
*MT-ATP6*	1	0.9	0	1	0	0
*MT-CO1*	1	0.9	1	0	0	0
*MT-ND4*	1	0.9	0	0	1	0
*MT-TF*	1	0.9	0	1	0	0
*PMPCA*	1	0.9	1	0	0	0
*Unknown*	16	15.1	9	1	3	3

*N* number of patients, *AD* autosomal dominant, *AR* autosomal recessive

Movement disorders were classified into hypokinetic and hyperkinetic [8]. Although ataxia and myoclonus are sometimes classified as hyperkinetic movement disorders, they are not considered classic extrapyramidal “movement disorders” and were considered separately. Hyperkinetic were further subdivided into three subgroups: dystonia, chorea-ballism and tremor. Dystonia, tremor and myoclonus were further defined, according to its respective recent classifications [9–11]. Moreover, ataxia was classified into pure cerebellar, spinocerebellar, sensory system involvement or mixed type [12, 13].

Clinical course was considered as a categorical variable, as follows: progressive, stable or ameliorative. Autonomy in daily life was also considered a categorical variable as follows: independent, minimal assistance, partially dependent (may cooperate in some tasks) and totally dependent.

Drug therapy was also valuated, and the clinical response was defined as no improvement vs clinical improvement.

Analysis of neurophysiological exams considered nerve conduction studies and electromyography. Evoked potentials were not routinely performed, and therefore, were not analyzed. Analysis of neuroimaging was defined trough categorical data: cerebral atrophy, cerebellar atrophy, brain stem atrophy, spinal cord atrophy, white matter hyperintensities, basal ganglia abnormalities (calcifications, iron deposition), nigro-striatal degeneration on 123I-FP-CIT SPECT or 18-F-DOPA PET, and evidence of stroke-like lesions.

Statistical analyses were conducted using MedCalc^®^ software, version 18.10.2. Two-tailed Fisher’s exact test was used for categorical associations. Continuous variables were analyzed by unpaired two-tailed Student’s *t*-test. Bonferroni’s correction for multiple tests was applied where appropriate. Statistical significance was set at a two-tailed *P* value of < 0.05. A “non-significant trend” was defined by a *P* < 0.05, however, not reaching formal statistical significance after Bonferroni’s correction.

## Results

### Features of PMD patients with movement disorders

Among the 764 symptomatic adult patients included in the Italian database who had a fully described clinical picture, 105 (13.7%, 56 females) were listed as suffering from different movement disorders. The movement disorders group had mean PMD onset of 38.6 ± 15.7 years (M = 37.4 ± 15.7; F = 39.6 ± 15.7, ns) and mean age at last evaluation of 55.1 ± 13.5 years, whereas among the patients without movement disorders the onset was at 36.3 ± 15.6 years and last evaluation at 49.2 ± 16.1 years (not significant differences after Bonferroni’s correction for two tests). Age at onset of movement disorders was 45 ± 18.1 (M 44.1 ± 17.8; F 45.7 ± 18.3, ns).

In 40/105, the onset of movement disorders coincided with PMD onset, in 9 preceded the diagnosis of PMD, while in 56 movement disorders developed after 13.7 ± 12.3 years from the PMD diagnosis. Mean duration of follow-up was 9.0 ± 7.2 years.

**Fig. 1:**
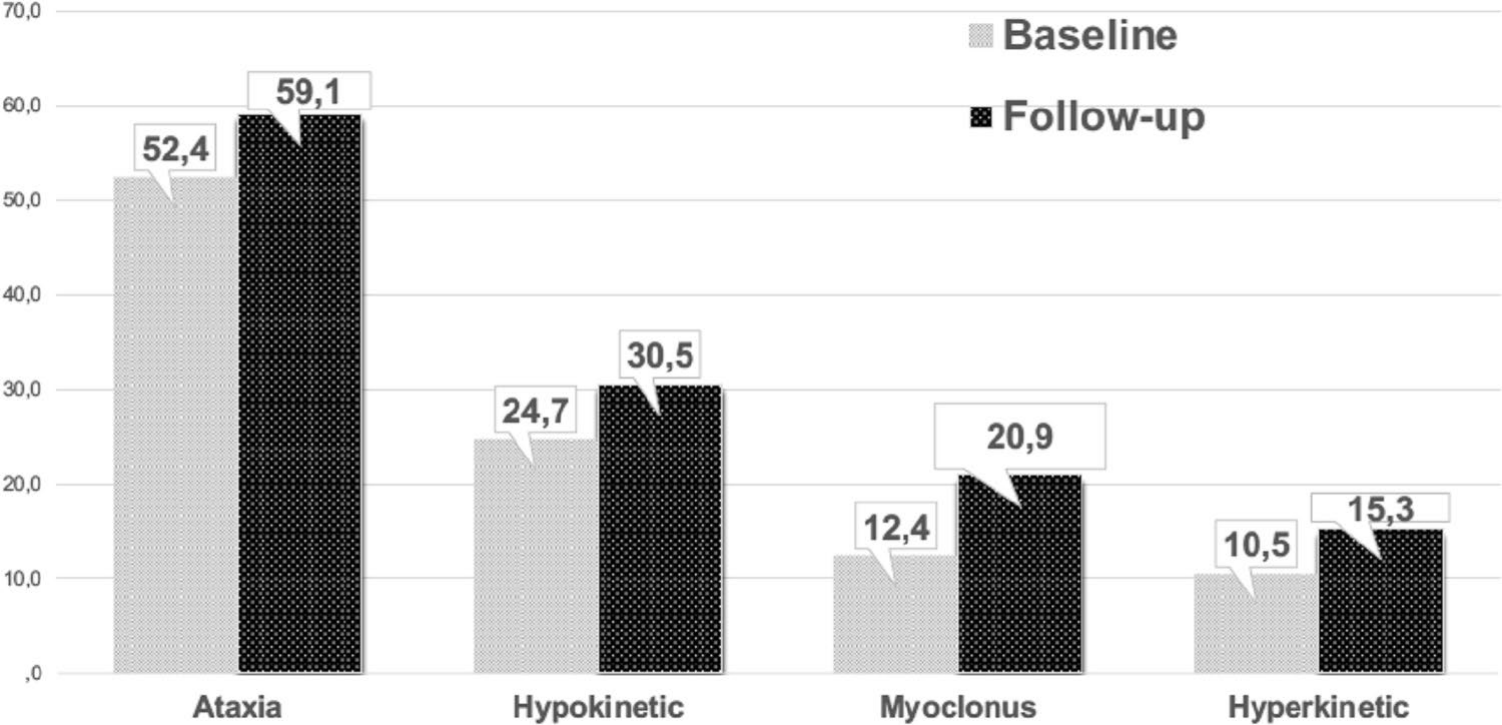
Prevalence of movement disorder phenotypes at baseline and last follow-up: percentages refers to proportions within the 105 patients who have a mitochondrial movement disorders

The most frequent phenotype at baseline was ataxia 55/105 (52.4%) followed by hypokinetic (26/105, 24.7%), hyperkinetic (11/105, 10.5%) movement disorders, and myoclonus (13/105, 12.4%) (Table 1 and Fig. 1).

Nineteen patients developed either ataxia or another movement disorder in the disease course, making the phenotype more complex: three showed myoclonus, six had a hypokinetic movement disorder, seven had ataxia and three a hyperkinetic movement disorder (one tremor and two dystonia) (Table 2).

Table 2 and e-Figure 1 report the genetic etiology. Overall, 42/105 patients harbored an mtDNA pathogenic variant: 9 mtDNA single deletion, the others mtDNA point pathogenic variants (mainly in the tRNAs) whereas 37 harbored a nuclear gene pathogenic variant and 10 multiple deletions (common genes excluded). In 16 patients, the molecular defect remains unknown. For the last two groups with unknown or unclear genetic defect, the diagnosis of PMD was based on clinical and instrumental findings (including, but not limited to, muscle biopsy) [14, 15].

**Table 3:** Genotype–phenotype correlation

	Movement disorders: yes (*n* = 105)	Movement disorders: no (*n* = 659)	
m.3243A > G pathogenic variant	8 (7.6%)	47 (7.1%)	n.s.
m.8344A > G pathogenic variant	17 (16.2%)	16 (2.4%)	** < 0.0001**
mtDNA LHON pathogenic variants	1 (0.9%)	154 (23.4%)	** < 0.0001**
mtDNA single deletion	9 (8.6%)	125 (19.0%)	**0.003**
nDNA: *OPA1* pathogenic variants	4 (3.8%)	25 (3.8%)	n.s.
nDNA: *POLG* pathogenic variants	23 (21.9%)	19 (2.9%)	** < 0.0001**
nDNA: *Twinkle* pathogenic variants	7 (6.7%)	23 (3.5%)	n.s.

The patients have been divided in two groups, with and without movement disorders. Genotypes with less than 25 patients have not been considered and are not shown. Significance levels after Bonferroni’s correction 0.007. Significant differences are represented in bold

*n.s.* not significant

Regarding the genotype–phenotype correlation, the pathogenic mtDNA m.8344A > G and *POLG* variants were significantly associated with the presence of a movement disorder (Table 3), while “LHON”-associated pathogenic variants and mtDNA single deletion were more commonly found in subjects who did not developed a movement disorder.

**Fig. 2:**
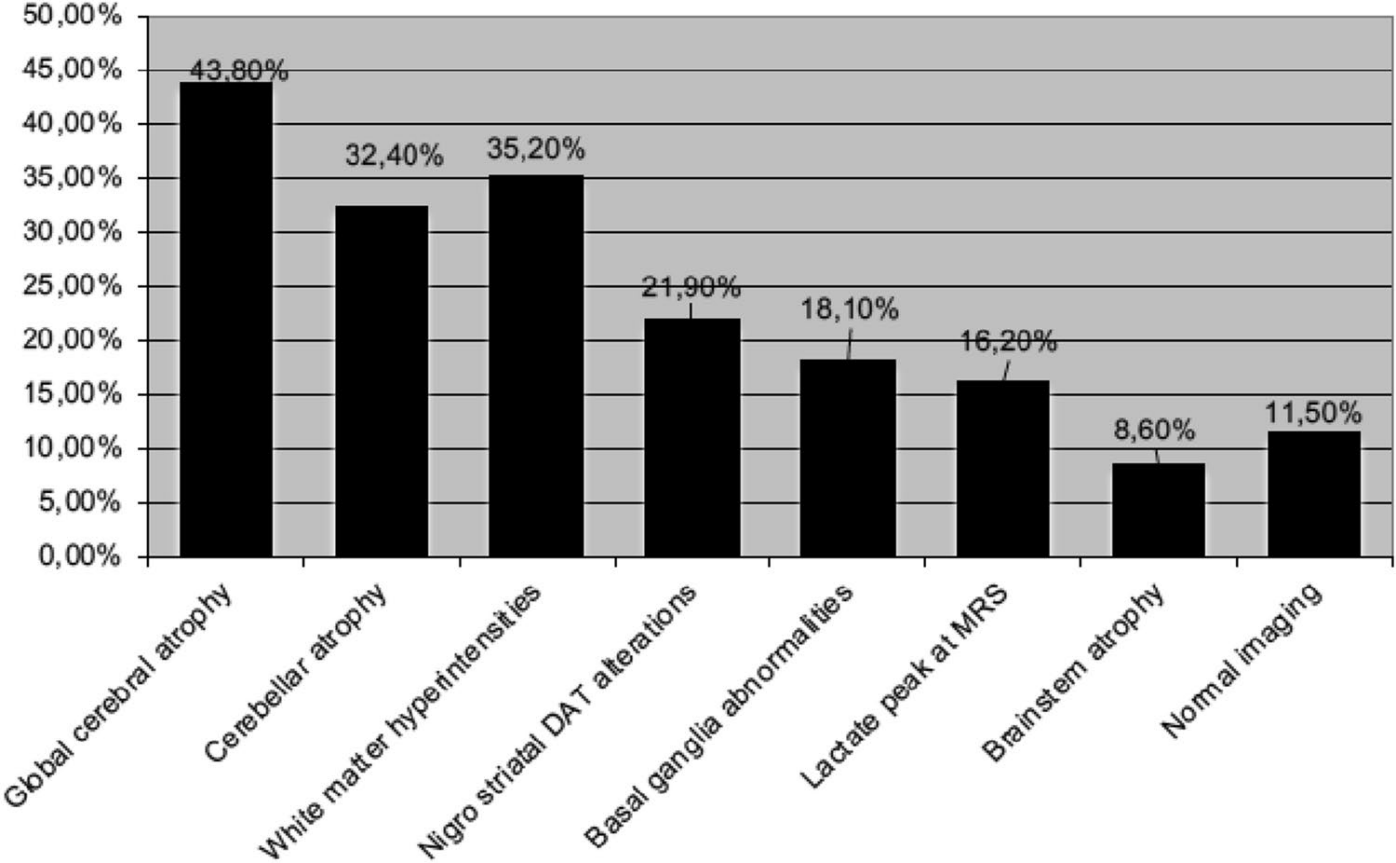
Neuroimaging features of patients with movement disorders

The most encountered MRI features were global cerebral atrophy (46/105; 43.8%), cerebellar atrophy (32.4%), white matter hyperintensities (35.2%), with normal MRI findings in 11.5% of cases (Fig. 2). A nigro-striatal alteration was detected in 21.9% of cases.

E-Table 1 shows MRI features and their association with specific genotypes. The only significant finding is a negative association between *POLG* variants and white matter hyperintensities. No associations between the m.3243A > G variant and white matter hyperintensities, between *TWNK* variants and cerebral atrophy, and *POLG* variants and a normal MRI were observed.

Furthermore, 44/105 (41.9%) patients had an axonal neuropathy (32 sensory and 12 sensory motor axonal neuropathy).

The clinical course was progressive in 81% and stable in 19%. At the last follow-up (mean value 9.0 ± 7.2 years), 22/105 (21%) patients were completely independent, 32/105 (30.5%) needed minimal assistance, 34/105 (32.4%) were partially dependent on a caregiver, and 17/105 (16.1%) were totally dependent on a caregiver.

### Hypokinetic movement disorder

A predominant hypokinetic movement disorder was the manifesting feature in 26/105, in 6 cases was the first clinical manifestation while in the remaining cases developed after a mean of 18.9 years (range 1–55) after PMD diagnosis (E-Table 2). All patients fulfilled the UK-PD Society brain bank criteria for parkinsonian syndrome [16]. Age at onset of the movement disorder was 62.7 ± 11.2 years (vs 48.3 ± 18.1 for PMDs) and mean follow-up was 8.9 ± 6.0 years.

Clinical course was progressive in 21/26 and stable in 5; unfortunately, UPDRS values were not collected. Six patients were completely independent, 14 needed minimal assistance, 4 were partially dependent on a caregiver and 2 totally dependent for daily activities. Dysphagia was observed in seven patients. Of the 26 patients, one received polytherapy with levodopa and dopamine agonist without improvement, 3 were not treated, and 22 received a monotherapy: 19 levodopa/dopa decarboxylase inhibitor up to 800 mg/day (18 with partial/full benefit, 1 without) and 3 dopamine agonists (pramipexole and rotigotine) with benefit. In a few patients, the clinical course was complicated by additional movement disorders. One levodopa-treated patient developed dyskinesia, a single case harboring an *OPA1* variant developed focal dystonia during disease course, and one *POLG* patient developed progressive ataxia.

The overall prevalence of hypokinetic movement disorders at the last follow-up was 30.5%. Six ataxic patients (four *POLG*, two *OPA1*) later developed a hypokinetic movement disorder during disease course, one with a poor response and another one with good response to levodopa therapy. E-Table 3 shows the genotype–phenotype correlation for hypokinetic movement disorders. *POLG* and *TWNK* pathogenic variants were significantly associated with hypokinetic movement disorders, whereas the opposite was observed for the LHON pathogenic variants.

Neuroimaging data mainly showed diffuse cerebral atrophy (46.2%), white matter hyperintensities 42.3%, basal ganglia alteration (15.4%). In 61.5%, nigro-striatal degeneration on 123I-FP-CIT SPECT or (18)F-dopa PET SPECT/PET alteration was detected.

Nerve conduction studies revealed an axonal sensory neuropathy in 4/26, axonal sensorimotor neuropathy in 2. EMG was myopathic in 20 and neurogenic in 2 (being normal in 4).

### Ataxia

Fifty-five patients (52.4%, 27 male) presented ataxia as main phenotype at the onset (Fig. 1). Age at diagnosis of MD was 36.7 ± 14.1 years, while age at onset of ataxia was 38.8 ± 16.4 and the mean follow-up 8.00 ± 6.7 years. Ataxia was the presenting features of MD in 34/55 (61.8%).

In 46/55 patients, clinical phenotype was defined as pure ataxia; in three cases, ataxia was associated with pyramidal signs, in four with myoclonus, in single cases with myoclonus and dystonia or with chorea and dystonia, respectively. Ataxia was cerebellar in 22/55, pure sensory ataxia in 9/55 (all with axonal neuropathy, sensory in seven and sensory motor in 2, mostly related to *POLG*) and spinocerebellar in 24/55.

Ataxic signs involved trunk limb and gait ataxia in 20/55, limb and gait in 16/55, only gait ataxia in 10/55 patients, gait and limb in 2, trunk and gait in 1, limb ataxia and trunk with gait ataxia in 3, respectively.

Twenty-three ataxic patients harbored nDNA pathogenic variants (16 *POLG1*, 2 *OPA1* and *TWNK*, 1 *AARS2*, *DARS2*, and *PMPCA*), 1 patient had multiple mtDNA deletions and 22 patients presented mtDNA variants. E-Table 4 shows genotype–phenotype correlations for ataxia: *POLG* and m.8344A > G pathogenic variants were significantly associated with ataxia, while the opposite was true for the LHON pathogenic variants.

Later in the disease course, nine ataxic patients developed other movement disorders: six had parkinsonism (4 *POLG*, 2 *OPA1*), three with mtDNA point pathogenic variants developed myoclonus. On the contrary, six patients with myoclonus and one *POLG* parkinsonism later developed ataxia (4 cerebellar, 2 mixed, 1 sensory), with an overall prevalence of ataxia at last follow-up of 62/105, 59.1% (Fig. 1).

Clinical course was progressive in 51 patients and stable in 4. Four patients were completely independent, 13 needed minimal assistance, 25 were partially dependent on a caregiver and 13 totally dependent for daily activities. SARA score was available for 33 patients: SARA at follow-up statistically deteriorated (16.8 ± 9.2 at last follow-up vs 10.8 ± 7.7 at baseline, *P* < 0.05).

Neuroimaging data revealed mainly global atrophy in 40%, cerebellar atrophy in 42%, brain stem atrophy and basal ganglia abnormalities in 14.5%, white matter hyperintensities in 29%, and lactate peak on spectroscopic MRI in 16%. Seven cases presented SPECT alterations (with parkinsonism in five). Spinal cord MRI revealed diffuse atrophy in three cases and abnormal signal in posterior columns in one DARS2 subject.

Nerve conduction studies revealed an axonal sensory motor neuropathy in 10, sensory axonal neuropathy in 22 and normal in 8 patients (data not available for 16 patients). EMG was myopathic in 12 cases and neurogenic in 16.

### Myoclonus

Myoclonus was the predominant movement disorder at onset in 13/105 patients (12.3%, Fig. 1), with a mean age at onset of 32.2 ± 11.8 years and a mean follow-up of 9.9 ± 7.8. Six patients showed myoclonus at baseline in association with other movement disorders. Three mtDNA patients developed myoclonus later, at follow-up, with a final prevalence of myoclonus at follow-up of 22/105 (20.9%).

Myoclonus was mainly associated with mtDNA pathogenic variants (10/13), mainly the m.8344A > G *MT-TK* (*P* < 0.01).

Myoclonus was generalized in 6/22 (27.3%), multifocal in 8/22 (36.4%), focal in 5/22 (22.8%), segmental in 1 (4.5%), not classified in 2 (9.0%). Considering the provoking factor, myoclonus was spontaneous in 9/22 (40.9%), spontaneous and reflex in 6/22 (27.3%) and spontaneous and action activated in 5/22 (22.8%).

Levetiracetam (1–4 g/day) was the most used drugs to treat myoclonus (50%), as monotherapy in nine cases, with good response. The other drugs used in our cohort were clonazepam (up to 2.5 mg/day), valproic acid, and in one case, piracetam.

### Hyperkinetic movement disorders

Eleven patients (10.5%, 8 females) presented a predominant hyperkinetic movement disorder: 6 tremor, 2 chorea-ballism, and 3 focal dystonia. Tremor was focal in four and segmental in two cases, and classified as mixed tremor in all cases. Age at diagnosis of PMD was 37.3 ± 13.2 years, while age at onset of movement disorder was 47.4 ± 14.4, with a mean follow-up of 12.9 ± 10.7 years. In all cases, the hyperkinetic movement disorder coincided or followed PMD onset (up to 28 years after PMD onset). No correlation between genotype and hyperkinetic movement disorder was observed.

Three patients developed minor hyperkinetic movement disorder at baseline in the context of another movement disorder, while two patients developed and hyperkinetic movement disorder at the follow-up. Therefore, the prevalence of hyperkinetic movement disorders at last follow-up was 16/105 = 15.3% (Fig. 1). Given the poor representation of hyperkinetic disorders, we have no statistical power in this category.

Considering the treatment, dystonia was treated with clonazepam, levodopa or botulinum toxin with poor response; one choreic patient was treated with tetrabenazine with improvement, and propranolol was used to treat tremor without success.

## Discussion

Among the 764 patients with adult-onset PMD, we identified 105 cases with a movement disorder during the disease course, estimating a minimum prevalence of movement disorders in PMD of 13.7%, which is higher compared to what has been observed in a recent study from Newcastle [4], most likely due to the fact we have included patients with ataxia and myoclonus.

In our cohort, ataxia was the commonest features being present in 62/105 (59.1%) of patients, often in association with other movement disorders, mainly myoclonus in patients harboring a *MT-TK* pathogenic variants or cases presenting parkinsonism and *POLG* pathogenic variants, making the clinical pictures complex. Similar data on prevalence of ataxia are reported in the UK-based Mitochondrial Research Center-funded cohort study, which estimates a frequency of ataxia of 65.2% [5].

Ataxia was associated with both *POLG* and m.8344A > G pathogenic variants. Ataxia was mostly cerebellar or spinocerebellar; pure sensory ataxia was found in 16% of ataxic patients, mainly in *POLG* pathogenic variants in the setting of a SANDO phenotype. Although our data on genotype–phenotype correlations did not reach a statistical association, ataxia was also frequently observed in *MT-TL1* patients, in Kearns Sayre syndrome and other mitochondrial or nuclear variants. Surprisingly, SPG7, which causes PMD trough impaired mtDNA maintenance, is not represented in our cohort despite ataxia and parkinsonism are commonly reported features [17, 18]. This could be related to referral of patients, as those harboring biallelic variants in *SPG7* may escape recording in the Italian Network National Registry.

Not surprisingly, myoclonus is commonly observed in mitochondrial encephalopathies, mainly in MERRF, as predominant features at onset or appearing at follow-up in ataxic patients, as already described [19]. On the other hand, hyperkinetic movement disorders (chorea, dystonia, ballism, and tics) are rarely observed in adults.

The most frequent MRI abnormalities were global cerebral or cerebellar atrophy, basal ganglia abnormalities and white matter hyperintensities, although we did not find statistically significant association in the genotype-neuroradiological approach.

Unfortunately, we cannot provide data on the pattern/distribution of cerebral or cerebellar atrophy, which may play an important role in the pathogenesis of movement disorders. Schergelman and co-workers [20] showed that cerebellar atrophy was more pronounced in PMD patients with movement disorders than in patients without, suggesting an important role of cerebellum. Intriguingly, this pattern of cerebellar atrophy was also found in PMD patients with parkinsonism in association with a volume increase in the putamen. On the contrary, PMD patients without movement disorders showed no cerebellar atrophy but had reduced caudate gray matter and superior medial gyrus volume. In our study, patients with parkinsonian syndrome showed frequently DAT imaging abnormalities; however, this finding should be carefully interpreted since it has been reported even without clinical manifest movement disorders [21]. In fact, Tzoulis and colleagues showed that despite severe mitochondrial alteration and neuronal loss in substantia nigra pars compacta (valued by dopamine transporter imaging and PET), many *POLG* patients did not show signs of clinical parkinsonism, probably by compensation of other brain structures such as thalamus and cerebellum. On the other hand, clinical parkinsonism in PMD can present with normal DAT findings [4].

Although under-represented in our series, given the high incidence of neuropathy in our cohort (44/105 = 42%), it is important to differentiate a truly hyperkinetic disorders from similar conditions such as pseudoathetosis or a neuropathic tremor: the latter are usual distal and associated with hypopallesthesia, while truly choreic movement can involve face and bulbar district.

Another important message delivered by our cohort study is to consider a mitochondrial etiology in parkinsonism or other movement disorders, especially when other mitochondrial “red flags” are associated; in these conditions, we recommend to screen patients for variants in mitochondrial nuclear genes (mainly *TWNK*, *OPA1*, *POLG1* and *SPG7)*. On the other hand, mtDNA primary pathogenic variants seem to be more linked with ataxia. L-DOPA therapy was effective in most patients, although the response to this therapy is variable in mitochondrial parkinsonism and asymmetric DAT-SPECT findings are considered predictor of L-DOPA response [22–24]. Levetiracetam was the most used and effective therapy to treat myoclonus, confirming previous observations on safety and efficacy [25–28]. In our cohort, in 30% of cases parkinsonism developed before the diagnosis of PMD. Age at onset of mitochondrial parkinsonism (in our cohort 62.7 ± 11.2) is usually earlier than idiopathic Parkinson disease (iPD). However, given that 56.25% of patients developed the parkinsonism after the age of 60 (E-Table 2), when iPD usually begins [29], a possible co-occurrence of PMD and iPD cannot be ruled out. The parkinsonian features, the nigro-striatal degeneration on 123I-FP-CIT SPECT, the high rate of L-DOPA response are also typical features of iPD and found in several patients of our cohort. Neuroimaging data did not allow us to differentiate between iPD and PMD: global cerebral atrophy, cerebellar atrophy, and even the spectroscopic lactate peak are detected in both iPD and mitochondrial parkinsonism [30–32]. Moreover, the group with parkinsonism onset before PMD did not show any peculiar clinical, genetic or imaging features. On the other hand, nigro-striatal vulnerability to mitochondrial dysfunction and phenotype complexity do not support iPD and PMD coexistence. Further studies are needed to identify—if any—clinical, biological and neuroimaging biomarkers of mitochondrial parkinsonism.

24.7% patients in our series (28.2% of hypokinetic movement disorders group) did not have a molecular diagnosis (unknown gene or multiple mtDNA deletions). In this group, the diagnosis of PMD was based on the presence of muscle, central nervous system, and multisystem involvement, biochemical and imaging results, and in case of biopsy the results of histology [33]. However, it must be considered that multiple mtDNA deletions and mitochondrial abnormalities on muscle biopsy (ragged red and COX negative fibers) can be found in other conditions, including iPD [30, 34] and are, therefore, not diagnostic. In these patients, with inconclusive genetic tests, a “possible mitochondrial diagnosis” [35] should be considered.

In conclusion, movement disorders in PMDs are not the most common findings in adult PMDs, but have a significant role on disease burden. The neurologists should be aware of the mitochondrial etiology of movement disorders, especially in the presence of mitochondrial red flags (i.e., diabetes, optic atrophy, epilepsy, myopathy) along with evocative MRI abnormalities (i.e., basal ganglia alterations or cerebellar atrophy), and consequently a mitochondrial genetic analysis should be considered according to the phenotype of the movement disorder.

## Supplementary Information

Below is the link to the electronic supplementary material.

Supplementary file1 Supplementary Figure 1: genotypes of patients with movement disorders (Doc 206 kb)

## Data Availability

Current article data are accessible from Michelangelo Mancuso, University of Pisa. In accordance with the data protection legislation in Europe (General Data Protection Regulation), to share the data of the Italian Network, it is necessary to stipulate an agreement between the University of Pisa and the applicant institution. Study data can be requested by contacting Michelangelo Mancuso (michelangelo.mancuso@unipi.it).

## Abbreviations

MDs: Primary mitochondrial diseases
MD: Primary mitochondrial disease
CNS: Central nervous system
MELAS: Mitochondrial encephalopathy, lactic acidosis, and stroke-like episodes
MERRF: Myoclonic epilepsy with ragged-red fibers
LHON: Leber hereditary optic neuropathy
MIRAS: Recessive mitochondrial ataxia syndrome
NARP: Neuropathy, ataxia, and retinitis pigmentosa
PET: Positron emission tomography
SPECT: Single-photon emission computed tomography
MRI: Magnetic resonance imaging
mtDNA: Mitochondrial DNA
POLG: Polymerase gamma
OPA1: OPA1 mitochondrial dynamin like GTPase
TWNK: Twinkle
SARA: Scale for assessment and rating of ataxia

## References

[1] Gorman GS, Chinnery PF, DiMauro S (2016). Mitochondrial diseases. Nat Rev Dis Prim.

[2] La Morgia C, Maresca A, Caporali L, Valentino ML, Carelli V (2020). Mitochondrial diseases in adults. J Intern Med.

[3] Musumeci O, Oteri R, Toscano A (2020). Spectrum of movement disorders in mitochondrial diseases. J Transl Genet Genomics.

[4] Martikainen MH, Ng YS, Gorman GS (2016). Clinical, genetic, and radiological features of extrapyramidal movement disorders in mitochondrial disease. JAMA Neurol.

[5] Lax NZ, Hepplewhite PD, Reeve AK (2012). Cerebellar ataxia in patients with mitochondrial DNA disease: a molecular clinicopathological study. J Neuropathol Exp Neurol.

[6] Christensen CK, Walsh L (2018). Movement disorders and neurometabolic diseases. Semin Pediatr Neurol.

[7] Ticci C, Orsucci D, Ardissone A, Bello L, Bertini E, Bonato I (2021). Movement disorders in children with a mitochondrial disease: a cross-sectional survey from the Nationwide Italian Collaborative Network of Mitochondrial Diseases. J Clin Med.

[8] Fahn S (2011). Classification of movement disorders. Mov Disord.

[9] Albanese A, Bhatia K, Bressman SB (2013). Phenomenology and classification of dystonia: a consensus update. Mov Disord.

[10] Bhatia KP, Bain P, Bajaj N (2018). Consensus Statement on the classification of tremors from the task force on tremor of the International Parkinson and Movement Disorder Society. Mov Disord.

[11] Zutt R, Van Egmond ME, Elting JW (2015). A novel diagnostic approach to patients with myoclonus. Nat Rev Neurol.

[12] Ashizawa T, Xia G (2016). Ataxia. Contin Lifelong Learn Neurol.

[13] Bodranghien F, Bastian A, Casali C (2016). Consensus paper: revisiting the symptoms and signs of cerebellar syndrome. Cerebellum.

[14] Parikh S, Karaa A, Goldstein A (2019). Diagnosis of possible’ mitochondrial disease: an existential crisis. J Med Genet.

[15] Witters P, Saada A, Honzik T (2018). Revisiting mitochondrial diagnostic criteria in the new era of genomics. Genet Med.

[16] Hughes AJ, Daniel SE, Kilford L, Lees AJ (1992). Accuracy of clinical diagnosis of idiopathic Parkinson's disease: a clinico-pathological study of 100 cases. J Neurol Neurosurg Psychiatry.

[17] De la Casa-Fages B, Fernández-Eulate G, Gamez J (2019). Parkinsonism and spastic paraplegia type 7: expanding the spectrum of mitochondrial Parkinsonism. Mov Disord.

[18] Pfeffer G, Pyle A, Griffin H (2015). SPG7 mutations are a common cause of undiagnosed ataxia. Neurology.

[19] Mancuso M, Orsucci D, Angelini C (2013). Phenotypic heterogeneity of the 8344A>G mtDNA “MERRF” mutation. Neurology.

[20] Schreglmann SR, Riederer F, Galovic M (2018). Movement disorders in genetically confirmed mitochondrial disease and the putative role of the cerebellum. Mov Disord.

[21] Tzoulis C, Tran GT, Schwarzlmüller T (2013). Severe nigrostriatal degeneration without clinical parkinsonism in patients with polymerase gamma mutations. Brain.

[22] Wilcox RA, Churchyard A, Dahl HH, Hutchison WM, Kirby DM, Thyagarajan D (2007). Levodopa response in Parkinsonism with multiple mitochondrial DNA deletions. Mov Disord.

[23] Miguel R, Gago MF, Martins J, Barros P, Vale J, Rosas MJ (2014). POLG1-related levodopa-responsive parkinsonism. Clin Neurol Neurosurg.

[24] Invernizzi F, Varanese S, Thomas A, Carrara F, Onofrj M, Zeviani M (2008). Two novel POLG1 mutations in a patient with progressive external ophthalmoplegia, levodopa-responsive pseudo-orthostatic tremor and parkinsonism. Neuromuscul Disord.

[25] Mancuso M, Galli R, Pizzanelli C, Filosto M, Siciliano G, Murri L (2006). Antimyoclonic effect of levetiracetam in MERRF syndrome. J Neurol Sci.

[26] Schinwelski M, Kierdaszuk B, Dulski J (2015). Changing phenotypic expression in a patient with a mitochondrial encephalopathy due to 13042G>A de novo mutation—a 5 year follow up. Metab Brain Dis.

[27] Su LJ, Wang YL, Han T (2018). Antimyoclonic effect of levetiracetam and clonazepam combined treatment on myoclonic epilepsy with ragged-red fiber syndrome with m.8344A>G mutation. Chin Med J (Engl).

[28] Altmann J, Büchner B, Nadaj-Pakleza A (2016). Expanded phenotypic spectrum of the m.8344A>G “MERRF” mutation: data from the German mitoNET registry. J Neurol.

[29] Ascherio A, Schwarzschild MA (2016). The epidemiology of Parkinson's disease: risk factors and prevention. Lancet Neurol.

[30] Henchcliffe C, Shungu DC, Mao X, Huang C, Nirenberg MJ, Jenkins BG, Beal MF (2008). Multinuclear magnetic resonance spectroscopy for in vivo assessment of mitochondrial dysfunction in Parkinson's disease. Ann N Y Acad Sci.

[31] O'Callaghan C, Hornberger M, Balsters JH, Halliday GM, Lewis SJ, Shine JM (2016). Cerebellar atrophy in Parkinson's disease and its implication for network connectivity. Brain.

[32] Filippi M, Sarasso E, Piramide N, Stojkovic T, Stankovic I, Basaia S, Fontana A, Tomic A, Markovic V, Stefanova E, Kostic VS, Agosta F (2020). Progressive brain atrophy and clinical evolution in Parkinson's disease. Neuroimage Clin.

[33] Witters P, Saada A, Honzik T, Tesarova M, Kleinle S, Horvath R, Goldstein A, Morava E (2018). Revisiting mitochondrial diagnostic criteria in the new era of genomics. Genet Med.

[34] Nicoletti V, Palermo G, Del Prete E, Mancuso M, Ceravolo R (2021). Understanding the multiple role of mitochondria in Parkinson's disease and related disorders: lesson from genetics and protein-interaction network. Front Cell Dev Biol.

[35] Parikh S, Karaa A, Goldstein A, Bertini ES, Chinnery PF, Christodoulou J, Cohen BH, Davis RL, Falk MJ, Fratter C, Horvath R, Koenig MK, Mancuso M, McCormack S, McCormick EM, McFarland R, Nesbitt V, Schiff M, Steele H, Stockler S, Sue C, Tarnopolsky M, Thorburn DR, Vockley J, Rahman S (2019). Diagnosis of 'possible' mitochondrial disease: an existential crisis. J Med Genet.

