# Supplementary material for: Adult-onset mitochondrial movement disorders: a national picture from the Italian Network
Source: J Neurol. 2021 Jul 14;269(3):1413–21. doi: 10.1007/s00415-021-10697-1 (PMC8857085; doi:10.1007/s00415-021-10697-1)

**E-Table 1. MRI features and their association with specific genotypes.** Genotypes with less than 5 patients in the movement disorder group have not been considered and are not shown. Significance levels after Bonferroni’s correction 0.002. N.s: not significant. Significant differences are represented in bold.

|  | **Cerebral Atrophy**  **(n = 46)** | **Cerebellar Atrophy**  **(n = 34)** | **Brainstem Atrophy**  **(n = 9)** | **Basal Ganglia Involvement**  **(n = 19)** | **White Matter hyperintensities**  **(n = 37)** | **Normal**  **(n = 12)** |
| --- | --- | --- | --- | --- | --- | --- |
| m.3243A>G  (n = 8) | 5 (62.5%)  n.s. | 5 (62.5%)  n.s. | 1 (12.5%)  n.s. | 1 (12.5%)  n.s. | 7 (87.5%)  *P* = 0.02 | 0  n.s. |
| m.8344A>G  (n = 13) | 7 (53.8%)  n.s. | 5 (38.5%)  n.s. | 0  n.s. | 1 (7.7%)  n.s. | 5 (38.5%)  n.s. | 3 (23.1%)  n.s. |
| mtDNA single deletion  (n = 9) | 1 (11.1%)  n.s. | 2 (22.2%)  n.s. | 2 (22.2%)  n.s. | 2 (22,2%)  n.s. | 4 (44,4%)  n.s. | 0  n.s. |
| nDNA: *POLG* pathogenic variants  (n = 23) | 8 (34.7%)  n.s. | 8 (34.7%)  n.s. | 1 (4.4%)  n.s. | 4 (17.4%)  n.s. | 5 (21,7%)  ***P* = 0.002** | 0  *P* = 0.03 |
| nDNA: *Twinkle* pathogenic variants  (n = 7) | 7 (100%)  *P* = 0.01 | 2 (28.6%)  n.s. | 1 (14.3%)  n.s. | 2 (28.6%)  n.s. | 3 (42.9%)  n.s. | 0  n.s. |

**E-Table 2:** Clinical features of the mitochondrial parkinsonism cohort.

| **Gene** | **Sex, MD age at onset (parkinsonism onset)** | **Signs** | **Clinical course** | **Autonomy** | **Therapy** | **Neuroimaging** | **Concomitant MD features** |
| --- | --- | --- | --- | --- | --- | --- | --- |
| C10Orf2 | F, 20 (75) | Bradikinesia, rest tremor, rigidity, postural instability | Progressive | Minimal assistance | L-DOPA 500 mg/day with improvment | Global cerebral atrophy, SPECT alteration, white matter hyperintensities | PEO, deafness |
| C10Orf2 | F, 45 (77) | Bradikinesia, rest tremor, rigidity, postural instability | Stable | Independent | No therapy | Global cerebral atrophy, SPECT alteration, white matter hyperintensities | PEO, sensory axonal neuropathy |
| C10Orf2 | F, 30 (63) | Bradikinesia, rest tremor, rigidity, postural instability | Stable | Independent | No therapy | Global cerebral atrophy, SPECT alteration, white matter hyperintensities | PEO, sensory axonal neuropathy |
| C10Orf2 | M, 55 (70) | Bradikinesia, rest tremor, rigidity, postural instability | Progressive | Minimal assistance | L-DOPA 500 mg/day with improvment | Global cerebral atrophy, Lactate peak, nigrosome alteration | PEO, OSAS |
| Multiple deletions | M, 20 (48) | Bradikinesia, rest tremor, rigidity, postural instability, mild upward gaze palsy | Progressive | Partly dependent | L-DOPA up to 1000 mg/day, partly effective and with dyskinesia | Parenchimal lactate peak, nigrostrotriatal SPECT alteration, global cerebral atrophy | Neuromuscolar disorders |
| Multiple deletions | M, 72 (71) | Bradikinesia, rigidity, camptocormia, | Progressive | Minimal assistance | L-DOPA, Pramipexole, without improvment | MRI normal, nigrostrotriatal SPECT alteration | Neuromuscolar disorders |
| Multiple deletions | F, 75 (75) | Bradikinesia, rigidity, rest tremor, postural instability, camptocormia | Progressive | Minimal assistance | L-DOPA up to 800 mg/day | Global cerebral atrophy, cerebellar atrophy, white matter hyperintensities, nigrostrotriatal SPECT alteration | PEO-plus, deafness, optic atrophy |
| Unknown | F, 78 (79) | Bradikinisia, rigidity, postural instability, | Progressive | Partly dependent | L-DOPA 300 mg/day with improvement | Global cerebral atrophy, nigrostrotriatal SPECT alteration | PEO, deafness, optic atrophy |
| POLG | F, 35 (59) | Bradikinisia, rigidity, rest tremor, postural instability, camptocormia, | Progressive | Minimal assitence | Pramipexole RP 2,1 mg/day with improvement | Nigrostriatal SPECT alteration | Diabetes, PEO, deafness |
| Unknown | F, 64 (64) | Bradikinisia, rigidity, postural instability, camptocormia | Progressive | Minimal assitence | Pramipexole RP 2,1 mg/day with improvement | Basal ganglia alteration, nisgrostriatal SPECT alteration | Neuromuscular disorder |
| Unknown | F, 45 (65) | Bradikinesia, rigidity, rest tremor, postural instability, camptocormia | Progressive | Independent | L-DOPA 300 mg/day with improvement | Nigrostriatal SPECT alteration | PEO, deafness, cardiomyopathy, diabetes |
| Multiple deletions | M, 32 (32) | Bradikinesia, rigidity, rest tremor, postural instability, camptocormia | Progressive | Minimal assitstance | L-DOPA up to 800 mg/day with improvment | Normal brain MRI | Neuromuscular disorder |
| POLG | M, 62 (62) | Bradikinesia, rigidity, postural instability, camptocormia | Progressive | Minimal assistance | Rotigotine 4 mg/day with improvment | White matter hyperintensities | Neuromuscular disorder |
| MT-ND4 | M, 34 (57) | Bradikinesia, rigidity, rest tremor, postural instability, camptocormia | Progressive | Independent | L-DOPA 750 mg/day with improvment | Nigrostriatal SPECT alteration | Optic atrophy |
| Multiple deletions | F, 65 (70) | Bradikinesia, rest tremor, postural instability, | Stable | Independent | L-DOPA 300 mg/day with improvment | Global cerebral atrophy, white matter hyperintensitie | PEO |
| Multiple deletions | F, 30 (58) | Bradikinesia, rigidity | Progressive | Minimal assistence | L-DOPA 300 mg/day with improvment | Normal Brain MRI | PEO |
| Single mtDNA deletion | M, 64 (67) | Bradikinesia, rigidity, rest tremor | Stable | Independent | L-DOPA 500 mg/day without improvment | White matter hyperintensities | PEO |
| Single mtDNA deletion | F, 65 (70) | Bradikinesia, rigity, rest tremor, camptocormia | Stable | Minimal assistance | L-DOPA 600 mg/day with improvment | White matter hyperintensities | PEO, deafness |
| Single mtDNA deletion | M, 30 (65) | Bradikinesia, rest tremor | Progressive | Minimal assistence | L_DOPA 450 mg/day with improvment | NA | PEO, sensory axonal neuropathy, retinopathy, deafness |
| POLG | F, 60 (61) | Bradikinesia, rigidity, rest tremor | Progressive | Minimal assistance | L-DOPA 600 mg/day with improvment | Nigrostriatal SPECT alteration | PEO, sensory axonal neuropathy |
| POLG | F, 28 (40) | Bradikinesia, rigidity, rest tremor | Progressive | Minimal assistance | L-DOPA 450 mg/day with improvment | Stroke like lesion | PEO, deafness, cardiomayopathy |
| POLG | M, 26 (47) | Bradikinesia, rigidity, rest tremor | Progressive, minimal assistance | Minimal assistance | NA | White matter hyperintensities | PEO, affective disorders |
| OPA1 | M, 50 (65) | Bradikinesia, rigidity, rest tremor, postural instability | Progressive | Total dependent | L-DOPA 600 mg/day with improvment | Global cerebral atrophy, basal ganglia abnormalities, white matter hyperintensities, nigrostriatal SPECT alteration | Optic atrophy, focal dystonia from age 72, axonal sensory motor neuropathy, PEO, affective disorders, diabetes |
| OPA1 | M, 65 (65) | Bradikinesia, rigidity, rest tremor, postural instability | Progressive | Total dependent | L-DOPA 400 mg/day with improvment | basal ganglia abnormalities, nigrostriatal SPECT alteration | PEO, optic atrophy, diabetes, affective disorders |
| C10Orf2 | M, 60 (71) | Bradikinesia, rigidity, postural instability | Progressive | Partly dependent | L-DOPA (no improvment) | Global cerebral atrophy, basal ganglia abnormalities | PEO, small fiber neuropathy, REM without atonia, deafness |
| POLG | F, 45 (56) | Bradikinesia, rigidity, postural instability | Progressive | Partly dependent | L-DOPA 300 mg/day with improvment | Global cerebral atrophy, white matter hyperintensities, lactate peak, SPECT alteration | PEO, axonal sensory motor neuropathy, ataxia, periodic limb movment, small fiber neuropathy |
| **Parkinsonism at follow up (during disease course)** | | | | | | | |
| POLG | M, 49 (49) | At baseline sensory ataxia; from age 73 bradikinesia, rigidity, postural instability, camptocormia | progressive | Minimal assistance | No efficacy L-DOPA or Rotigotine | Nigrostriatal SPECT alteration | Sensory axonal neuropathy, PEO, deafness |
| POLG | F, 31 (35) | At baseline sensory ataxia; from age 51, bradykinesia rigidity camptocormia | progressive | Minimal assistance | No therapy | White matter iperintensities, basal ganglia abnornamlities | Sensory axonal neuropathy, PEO, deafness, affective disorders |
| POLG | F, 31 (35) | At baseline sensory ataxia; from age 51 bradikinesia, rest tremor, rigidity | Progressive | Minimal assistance | No therapy | Basal ganglia abnormalities, SPECT alteration | Sensory axonal neuropathy, PEO, deafness. |
| OPA1 | M, 55 (55) | At baseline ataxia (mixed, sensory and cerebellar); from age 69 bradikinesia, rigidity, rest tremor, instability | progressive | Total dependent | L-DOPA 400 mg/day with improvment | Cerebral and cerebellar atrophy, basal ganglia abnormalities, white matter hyperintensities lactate peak, SPECT alteration | Optic atrophy, sensory axonal neuropathy, RBD, deafness, PEO, dementia |
| OPA1 | F, 45 (45) | At baseline ataxia (mixed, sensory and cerebellar); from age 72 bradikinesia and rigidity | progressive | Total dependent | No therapy | Cerebral and cerebellar atrophy, basal ganglia abnormalities, white matter hyperintensities lactate peak, SPECT alteration | Optic atrophy, PEO, Sensory motor axonal neuropathy, dementia, deafness |
| POLG | M, 30 (40) | At baseline sensory ataxia, frpm age 67 bradikinesia and tremor | progressive | Total dependent | No therapy | Cerebral and cerebellar atrophy, SPECT alteration | Sensory axonal neuropathy, PEO, restless leg syndrome, respiratory failure |

**E-Table 3. Genotype-phenotype relationship: hypokinetic movement disorders.** Significance levels after Bonferroni’s correction 0.007. Significant differences are represented in bold.

|  | **Hypokinetic disorders:**  **Yes (n = 26)** | **hypokinetic disorders:**  **No (n = 738)** |  |
| --- | --- | --- | --- |
| m.3243A>G | 0 | 53 (7.2%) | n.s. |
| m.8344A>G | 0 | 33 (4.5%) | n.s. |
| mtDNA LHON pathogenic variants | 0 | 154 (20.9%) | **0.005** |
| mtDNA single deletion | 3 (11.5%) | 131 (17.8%) | n.s. |
| nDNA: *OPA1* pathogenic variants | 2 (7.7%) | 27 (3.7%) | n.s. |
| nDNA: *POLG* pathogenic variants | 6 (23.1%) | 36 (4.9%) | **0.002** |
| nDNA: *Twinkle* pathogenic variants | 5 (19.2%) | 25 (3.4%) | **0.002** |

**E-Table 4. Genotype-phenotype relationship: ataxias.** Significance levels after Bonferroni’s correction 0.007. Significant differences are represented in bold.

|  | **Ataxia:**  **Yes (n = 55)** | **Ataxia:**  **No (n = 709)** |  |
| --- | --- | --- | --- |
| m.3243A>G | 8 (14.5%) | 47 (6.6%) | n.s. |
| m.8344A>G | 7 (12.7%) | 10 (1.4%) | **<0.0001** |
| mtDNA LHON pathogenic variants | 0 | 154 (21.7%) | **<0.0001** |
| mtDNA single deletion | 3 (5.4%) | 130 (18.3%) | 0.01 |
| nDNA: *OPA1* pathogenic variants | 2 (3.6%) | 27 (3.8%) | n.s. |
| nDNA: *POLG* pathogenic variants | 16 (29.1%) | 26 (3.7%) | **<0.0001** |
| nDNA: *Twinkle* pathogenic variants | 2 (3.6%) | 28 (3.9%) | n.s |

Supplementary Table: online 50-item Google Form®-based questionnaire

|  | Question | Answers |
| --- | --- | --- |
| 1 | Patient code |  |
| 2 | Sex |  |
| 3 | Date of birth |  |
| 4 | Referring physician |  |
| 5 | Gene |  |
| 6 | Inheritance |  |
| 7 | MD's age of Onset |  |
| 8 | Movement disorder age of onset |  |
| 9 | Follow up duration |  |
| 10 | Predominant movment disorder at onset |  |
| 11 | Secondary movment disorder at onset |  |
| 12 | If a rigid hypokinetic disorder was present at the onset, report the clinical features: | Bradikinisia, rigidity, rest tremor, postural instability, camptocormia |
| 13 | Dystonia distribution (if present) | Generalized, multifocal, focal, segmental |
| 14 | Chorea ballism distribution (if present) | Generalized, multifocal, focal, segmental |
| 15 | Myoclonus distribution (if present) | Generalized, multifocal, focal, segmental |
| 16 | Myoclonus provoking factor (if present) | Spontaneous, reflex, action activated |
| 17 | Classify tremor (if present) | Rest tremor, action tremor, mixed |
| 18 | Tremor distribution (if present) | Generalized, multifocal, focal, segmental |
| 19 | Ataxia phenotype (if present) | Cerebellar, spinocerebellar, pure sensory ataxia |
| 20 | Ataxia signs inolved | Trunk, limb, gait, |
| 21 | Did the patient have any other movement disorders during the course of the disease? | Yes/No |
| 22 | What other movement disorders were present during the disease course? |  |
| 23 | Age of onset of the movement disorder at follow up |  |
| 24 | If a rigid hypokinetic disorder was present at follow up, report the clinical features: | Bradikinisia, rigidity, rest tremor, postural instability, camptocormia |
| 25 | Dystonia distribution (if present, at follow) | Generalized, multifocal, focal, segmental |
| 26 | Chorea ballism distribution (if present, at follow) | Generalized, multifocal, focal, segmental |
| 27 | Myoclonus distribution (if present, at follow) | Generalized, multifocal, focal, segmental |
| 28 | Myoclonus provoking factor (if present, at follow) | Spontaneous, reflex, action activated |
| 29 | Classify tremor (if present, at follow) | Rest tremor, action tremor, mixed |
| 30 | Tremor distribution (if present, at follow) | Generalized, multifocal, focal, segmental |
| 31 | Ataxia phenotype (if present, at follow) | Cerebellar, spinocerebellar, pure sensory ataxia |
| 32 | Ataxia signs inolved (at follow up) | Trunk, limb, gait |
| 33 | Clinical course | progressive, stable or ameliorative |
| 34 | SARA score at baseline, if available |  |
| 35 | SARA score at follow up, if available |  |
| 36 | Autonomy in daily life | Independent, minimal assistance, partially dependent (may cooperate in some tasks) and totally dependent |
| 37 | Has drug therapy been set up to control the movement disorder? | Yes/No |
| 38 | Type of therapy | Monotherapy / polytherapy |
| 39 | Prescribed drugs |  |
| 40 | Which drugs were used with clinical improvement? |  |
| 41 | About the drug used with clinical improvment, reports the dosage |  |
| 42 | Neuroimaging | Cerebral atrophy, cerebellar atrophy, brain stem atrophy, spinal cord atrophy, white matter hyperintensities, basal ganglia abnormalities (calcifications, iron deposition), nigrostriatal degeneration on 123I-FP-CIT SPECT or 18-F-DOPA PET, and evidence of stroke-like lesions. |
| 43 | Nerve conduction studies | Normal, sensory neuropathy, sensory motor neuropathy, motor neuropathy |
| 44 | Electromyography | Normal, myopathic, neuropathic |
| 45 | For pediatric patients at the onset of movement disorder (<16 years), specify if there is a speech disorder | Yes/No |
| 46 | Describe the speech disorder |  |
| 47 | For pediatric patients, describe if there is a neurodevelopmental disorder associated with movement disorder (e.g., intellectual disability, autism spectrum disorder, ADHD) |  |
| 48 | Dysfagia | Yes/No |
| 49 | Other MD clinical features |  |
| 50 | Note |  |

E-Figure 1: genotypes of patients with movement disorders
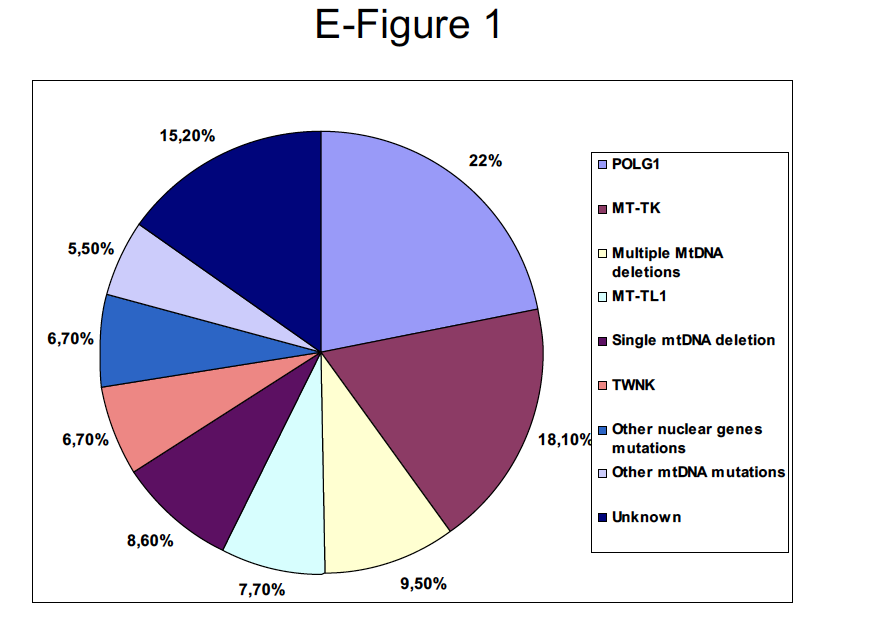

Supplement: Supplementary file 1 — Supplementary file1 Supplementary Figure 1: genotypes of patients with movement disorders (Doc 206 kb) [file 415_2021_10697_MOESM1_ESM.doc]
